# Fabrication of a Polycaprolactone/Chitosan Nanofibrous Scaffold Loaded with *Nigella sativa* Extract for Biomedical Applications

**DOI:** 10.3390/biotech12010019

**Published:** 2023-02-12

**Authors:** Qasim Shakir Kahdim, Najmeddine Abdelmoula, Hassan Al-Karagoly, Salim Albukhaty, Jabbar Al-Saaidi

**Affiliations:** 1 College of Basic Education, University of Babylon, Babylon 51002, Iraq; 2 Laboratory of Multifunctional Materials and Applications (LaMMA), LR16ES18, Faculty of Sciences of Sfax, University of Sfax, BP 1171, Sfax 3000, Tunisia; 3 College of Veterinary Medicine, University of Al-Qadisiyah, Al-Diwaniyah 58002, Iraq; 4 Department of Chemistry, College of Science, University of Misan, Maysan 62001, Iraq; 5 College of Medicine, University of Warith Al-Anbiyaa, Karbala 56001, Iraq

**Keywords:** *Nigella Sativa*, chitosan, electrospinning, polycaprolactone, biocompatibility

## Abstract

In this study, biocompatible electrospun nanofiber scaffolds were produced using poly(-caprolactone (PCL)/chitosan (CS) and *Nigella sativa* (NS) seed extract, and their potential for biomedical applications was investigated. Scanning electron microscopy (SEM), Fourier transform infrared spectroscopy (FTIR), total porosity measurements, and water contact angle measurements were used to evaluate the electrospun nanofibrous mats. Additionally, the antibacterial activities of *Escherichia coli* and *Staphylococcus aureus* were investigated, as well as cell cytotoxicity and antioxidant activity, using MTT and DPPH assays, respectively. The obtained PCL/CS/NS nanofiber mat was observed by SEM to have a homogeneous and bead-free morphology, with average diameters of 81.19 ± 4.38 nm. Contact angle measurements showed that the wettability of the electrospun PCL/Cs fiber mats decreased with the incorporation of NS when compared to the PCL/CS nanofiber mats. Efficient antibacterial activity against *S. aureus* and *E. coli* was displayed, and an in vitro cytotoxic assay demonstrated that the normal murine fibroblast cell line (L929 cells) remained viable after 24, 48, and 72 h following direct contact with the produced electrospun fiber mats. The results suggest that the PCL/CS/NS hydrophilic structure and the densely interconnected porous design are biocompatible materials, with the potential to treat and prevent microbial wound infections.

## 1. Introduction

Electrospinning is an efficient and practical method that utilizes a powerful electric force to move polymeric solutions to create micro- and nanofibers. Surface tension is deformed by the high voltage of an electrically conductive fluid in the fabrication of fibers, which have recently been investigated for their potential in biomedicine, such as in wound treatment and tissue engineering [1,2,3,4,5]. In practical electrospinning, a variety of variables, including melt or solution quality, electrospinning device parameters, and environmental variables, might influence the shape and characteristics of the resulting fibers [6,7]. However, to understand the effects of such complicated parameters on nanofibers when using novel polymers, composites with different mixes, and solvent combinations for electrospinning, extensive research is needed [8,9]. PCL, a synthetic polymer with FDA approval and with considerable mechanical and biocompatibility properties, has been frequently used as a polymer in electrospinning techniques for biomedical applications [10,11,12,13].

Chitosan electrospun nanofibers have gained a great deal of attention in the fields of fibrous wound healing and tissue engineering due to their unique properties, including biocompatibility, biodegradability, antibacterial activity, nontoxicity, antifungal activity, and drug-loading capacity [14,15]. Numerous investigations have been conducted on the electrospinning of chitosan and PCL in conjunction with other polymers or bioactive compounds for use in tissue engineering and wound dressings. Medical plant extracts have been utilized as a traditional treatment for wounds for decades due to their therapeutic effects, which include their antibacterial, antioxidant, anti-inflammatory, and wound-healing effects [16,17]. Nanofibers containing naturally derived bioactive materials are beneficial because of their high specific surface-area-to-volume ratio and extremely porous mesh, which offers a range of advantages for the treatment of both chronic and acute wounds compared to traditional dressings [18]. *Nigella sativa* (NS) is considered to be one of the most promising herbal products due to the presence of numerous powerful bioactive components, such as thymoquinone [19]. NS has been used for centuries in traditional medicine to treat skin disorders and as an analgesic, liver tonic, diuretic, digestive, anti-diarrheal, and antibacterial treatment [20,21,22,23,24,25]. It has several active ingredients and properties, including antioxidant and anti-inflammatory agents, anti-cancerogenic agents, antimicrobials, and immunostimulants [26,27,28]. Major nutrients in NS include carbohydrates, proteins, alkaloids, vitamins, and minerals. Thymoquinone, which makes up around 45% of its essential oils and is well known for its pharmacological and therapeutic properties, is one of the most prominent components of this plant [29,30]. *N. sativa* plant extracts and their natural compounds, used in nanoformulations, have demonstrated high activity in the management of wounds and thus can be assumed as future pharmaceutical drugs for prospective application as a wound dressing material. Shiva Teilaghi et al. evaluated an electrospun solution of zein and black seed (Nigella sativa) oil at three different oil concentrations of 5, 10, and 15% *w/v* in advanced drug delivery applications [31]. Fatemeh Kalhori et al. explored innovative electrospun mats that incorporated NS oil and polyacrylonitrile as a sustained-release nano-bandage to treat rheumatoid arthritis [32]. However, few investigations have focused on the electrospinning of Nigella sativa extract for application in antimicrobial wound dressings. In a recent study, Aras et al. [33]. performed a variety of experiments on the loading of Nigella sativa oil into a polyurethane nanofibrous mat for prospective application as a wound dressing material.

The objective of the current work was to combine the benefits of NS extract with CS and PCL to create a biocompatible nanofibrous mat that was inexpensive, non-toxic, and had antioxidant and antibacterial properties, which could be used as a wound dressing material. In the study, an NS-extract-loaded electrospun PCL/CS nanofibrous mat was investigated for potential use as an antioxidant and antibacterial wound dressing material. The produced mat was characterized by scanning electron microscopy (SEM). FTIR spectroscopy was used to identify the functional groups based on the peak values in the IR present in the native sample. The hydrophilicity of the scaffold was characterized by the surface contact angle test, and the scaffold’s porosity was also investigated. The cytotoxicity of the PCL/CS/NS nanofibrous mat was examined using the L929 cell line, as well as its antioxidant and antibacterial effects on strains of bacteria *E. coli* and *S. aureus*.

## 2. Materials and Methods

### 2.1. Material

All chemicals used were of analytical grade and were used as received, without any further purification. They were obtained from Sigma-Aldrich as follows: (PCL) Mn ∼80 000, chitosan (CS, molecular weight = 120,000 Da, degree of deacetylation = 85%), phosphate-buffered saline (PBS), Dulbecco’s modified Eagle’s medium (DMEM), fetal bovine serum (FBS), penicillin, streptomycin, dimethyl sulfoxide (DMSO), MTT, acetic acid, and formic acid.

The NS seeds, as raw plant materials, were acquired from a local market in Hilla City, Babylon, Iraq, in February 2022, and were authenticated by the College of Agriculture at the University of Babylon in Iraq. Nigella sativa was extracted following the technique described by H Mahmoudvand et al. [34], with some modifications.

### 2.2. Gas Chromatography–Mass Spectrometry (GC–MS) Analysis

Using a Perkin Elmer GC/MS-QP2 system and a Restek5, RT*R- (30 m × 0.215 mm) capillary column, the ethanolic extract of N. sativa was examined. The extract (1 mg/mL) was reconstituted in methanol before being injected into 1 μL at a split ratio of 20:1, with 99.9% helium gas as the carrier gas. The oven was gradually heated to 280 °C for 10 min, starting at 60 °C for 5 min, with the inflator at 250 °C. The mass-spectral database (NIST and WILLEY library) connected to the GC/MS system to obtain spectral configurations was used to identify the chemicals [35,36].

### 2.3. Production of the Electrospun PCL, CS, and NS Nanofibrous Mats

First, various mixtures of PCL/CS and NS extract with different ratios and concentrations were selected for the electrospinning process. The best combination, with an NS/PCL/CS ratio of 2/3/1, was chosen.

The first solution, the NS stock solution, was made by dissolving 0.1 g of NS extract in 100 mL of absolute ethanol (96%). Then, each 1 mL of this stock was supplemented with 5 μL of Tween 80.

A mixture of PCL (10% *w*/*v*) and CS (3% *w*/*v*) was made by dissolving 1 g of PCL and 0.3 g of CS in 10 mL of glacial acetic acid and formic acid at a 30:70 ratio, followed by stirring overnight. The mixtures were stirred for 4 h before electrospinning, followed by 20 min of ultrasonication. The PCL/CS solution was then combined with 7% *w/v* NS solution (5 mL), and the mixture was stirred for 3 h. Once these parameters were in place, the flow rate for electrospinning was set at 0.5% per hour, the applied voltage was set at 18 kilovolts, the tip–collector distance was set at 12 cm, and the drum collector’s rotation speed was fixed at 700 rpm [37].

### 2.4. Characterization of the PCL/CS/NS Nanofibrous Scaffold

#### 2.4.1. Morphological Analysis Using SEM

SEM (MIRA TESCAN, Czech Republic) was used to examine the morphology of the composite nanofibrous scaffold at a 15 kV accelerating voltage. The scaffolds were coated with gold before imaging using a sputter coater with a 15 kV acceleration voltage and a magnification scale of 100,000× *g*. Scaffold fiber diameters were calculated using image analysis software based on SEM images at 5000× *g* magnification (ImageJ, U.S. National Institutes of Health, Bethesda, Maryland, USA).

#### 2.4.2. Infrared Spectroscopy with Fourier Transform

PCL/PLA/NS nanofibers were obtained by combining 1 mg of sample with 100 mg of KBr of NS and using FTIR spectroscopy to analyze the structure and chemical makeup of the nanofiber, as well as any potential interactions between the extract and polymer in the nanofiber formations. In the 500–4000 cm^−1^ region, sample spectra were captured.

#### 2.4.3. Water Contact Angle and Mechanical Properties

The water contact angle and mechanical features of the scaffolds were assessed.

The contact angle was dynamically calculated using the Wilhelm plate approach [38]. The hydrophilicity or hydrophobicity of the sample was tested and evaluated using water with a surface tension of 72 dyn/cm. The liquid in the container increased until the metal plate’s intended surface was completely submerged in water, at which point it began to descend. We determined the porosity of the fibers by weighing the samples and soaking them in PBS for 24 h. The weight was once more measured after the surface water and nanofiber web were eliminated from the solution. The nanofibrous scaffold’s porosity was determined by the volume of liquid that it could retain.

### 2.5. In Vitro Cell Culture Studies

The MTT test was carried out to identify the cytoprotective criteria of PCL/CS/NS nanofibrous mats under conditions of oxidative stress. To encourage cell adhesion on the nanofiber surface, 1 cm^2^ nanofibrous mats were sterilized overnight in a laminar flow hood under UV light for both the top and bottom surfaces. After being rinsed with distilled water and PBS to remove any remaining solvent, they were then submerged in DMEM overnight. Cells from L929 were cultured in DMEM supplemented with 10% FBS and 1% antibiotics at 37 °C and 5% carbon dioxide. The L929 cells were trypsinized and seeded at 1 × 10^5^ cells per well onto the nanofibrous mat once they had attained % confluence. They were then incubated at 37 °C and 5% CO_2_. The MTT assay was used to test cell viability on the nanofibrous mats for 24, 48, and 72 h time periods.

### 2.6. Antibacterial Activity

The Kirby–Bauer disk diffusion method to test antibacterial activity (Humphries et al., 2018) [39] was considered a suitable method for evaluating the antibacterial activity of PCL/CS/NS nanofibers. Briefly, five bacterial colonies of *S. aureus* (ATCC 29213) and *E. coli* (ATCC 35218) were used to collect sterile inoculating loops, which were then suspended in 2 mL of sterilized PBS. By diluting the bacterial suspension with sterile PBS, the turbidity was already reduced to a 0.5 McFarland level. Inoculum channels were populated with sterile swabs. Bacterial swabs were inoculated into plates of Muller–Hinton agar. To disperse the nanofibers, we dissolved 0.1 mg PCL/CS/NS nanofibers in 1 mL of distilled water. Before use, the suspension was sonicated for 10 min. The standard was impregnated with 35 µL of the PCL/CS/NS nanofiber suspension, chitosan suspension, *Nigella sativa* extract (NS), distilled water (as a negative control), and antibiotic (as a positive control).

### 2.7. Activity of DPPH Radical Scavenging

This test was performed according to Blois’s description (1958) [40]. A total of 0.025 g/L of DPPH was dissolved in methanol. Dimethyl sulfoxide (DMSO) was used to dilute various concentrations of chitosan (CS), *Nigella sativa* (NS), nanofibers (PCL/CS/NS), and ascorbic acid (as a control) to create a sample solution. Following the addition of 5 µL of the sample solution to each well of a 96-well plate, 195 µL of the DPPH working suspension was pipetted. The reaction took place at room temperature for 20 min, and the solution’s absorbance at 515 nm was determined. The free radical scavenging activity of each fraction was assessed by comparing its absorbance to that of a blank solution (no sample). The following equation was used to calculate the percentage of inhibition, representing the capacity to scavenge DPPH radicals.

$$DPP\;Scavenging\;Activity\left(\%\right)=(A_{0\;}-A_{1})/A_{0}*100$$

*A*_0_ refers to the absorbance value of the control and *A*_1_ represents the absorbance value of the test sample.

## 3. Results and Discussion

### 3.1. GC–MS Analysis

**Figure 1:**
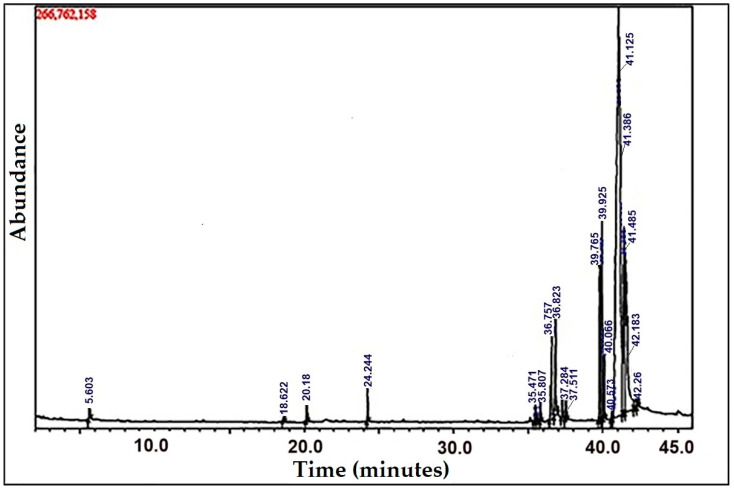
GC–MS chromatogram of the ethanol extract of *Nigella sativa* seeds.

Multiple peaks were detected in the GC–MS analysis of the ethanolic extract of *N. sativa* seeds (Figure 1). A repository of the recognized component spectra from the GC–MS library was used to determine the chromatogram peaks. According to GC–MS profiling, the extract contained 20 major elements. The discovered compounds are listed in Table 1, along with their peak area (%), retention time, chemical formula, and molecular weight. From these 20 compounds, six elements had a high peak percentage, including hexadecanoic acid–methyl ester, undedecanoic acid, L-ascorbic acid 2,6-dihexadecanoate, 9,12-octadecadienoic acid-(Z, Z)–methyl ester, 10,13-eicosadienoic acid, 9-octadecenoic acid (Z), 9,12-octadecadienoic acid, and ethyl ester (Table 1). Hexadecanoic acid–methyl ester and 10,13-eicosadienoic acid–methyl ester are natural acids with antifungal and antibacterial properties, particularly against *E. coli and Staphylococcus aureus* [41]. Moreover, L-(+)- ascorbic acid 2,6-dihexadecanoate is an essential molecule that functions as an antioxidant, cardiovascular protectant, cancer-preventative, and flavoring agent [42]. Meanwhile, 9,12-octadecadienoic acid (Z, Z)–methyl ester has analgesic, anti-inflammatory, and ulcerative characteristics [43]. This polyunsaturated fatty acid is found in many plant glycosides, including *Nigella sativa* [44]. Among their various roles in the body, polyunsaturated fatty acids play a key role in anti-inflammation, antioxidant, and anti-atherosclerotic activities via the regulation of vascular hemodynamics [45].

### 3.2. Morphology of Nanofibers

**Figure 2:**
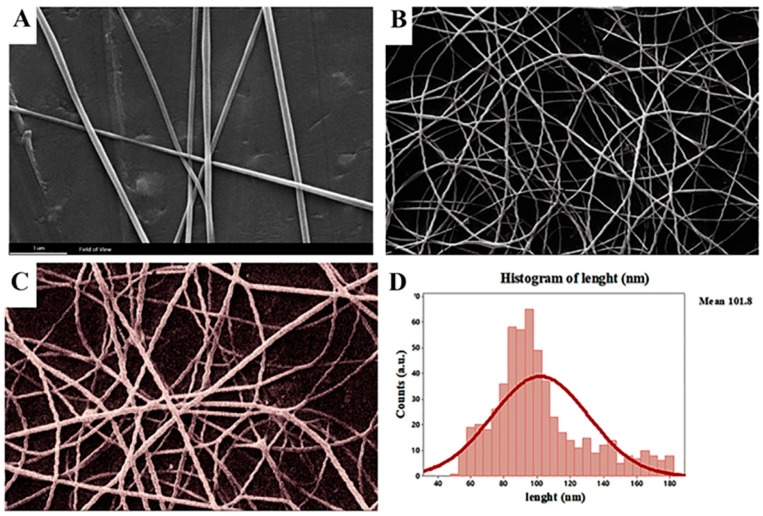
Electrospun PCL/CS/NS (mean diameter 81.19 ± 4.38 nm) in a SEM photograph: (**A**) PCL (1 µm); (**B**) PCL/CS/NS (1 µm); (**C**) PCL/CS/NS (500 nm); (D) The diameter size distributions.

SEM micrographs showed the prepared PCL/CS/NS at various weight ratios. From Figure 2, the nanofiber mat had a more uniform and thinner texture, demonstrating that a uniformly favorable solution viscosity was achieved during electrospinning by using the optimized operating parameters. The current study results revealed nanofibers of a diameter between 40 ± 2.03 and 180 ± 2.16 nm, with an average of 101.85 ± 4.38 nm. The morphology of the prepared electrospun nanofibers was directly influenced by parameters such as the voltage, flow rate, and the distance from the tip to the collector.

CS and NS have many polar groups, such as –NH2 and –COOH, which have the potential to carry positive or negative charges and create a polyanion–polycation complex. A larger charge density on the surface would result in more elongation forces being applied to the released jet [46]. Additionally, the increased charge density may increase the jet bending instability, resulting in a reduced fiber diameter [47]. Incorporating N. sativa into PCL/CS nanofibers resulted in shifting the fibers’ diameter and diameter distribution to lower values, which was in agreement with other previous reports [33].

The mechanical properties of PCL nanofibers have been enhanced through the addition of various fillers, including nanosilicates, graphene, cellulose nanocrystals, and Ag nanoparticles [48,49]. The mechanical strength of PCL nanofibers for biomedical applications has also been improved by blending them with natural or synthetic polymers [50]. Wang et al. (2021) [51] reported that adding natural polymers such as chitosan, alginate, and lignin to PCL nanofibers can improve their structural integrity, and hence their functionality. Since thymol was the active component of the NS extract, it stands to reason that this extract could serve as a plasticizer and regulator of the polymer chains, resulting in a reduction in the diameter of the nanofibers [52].

The shear viscosity of the spinning solution is often believed to be the key variable of the fiber diameter [53]. When the viscosity is too low, polymeric fibers and droplets of the material (electrospray) may be interrupted, while, when the viscosity is too high, the polymeric material cannot be extruded [54]. The needed minimum viscosity threshold varies with the molecular weight of the polymer and the type of solvent being employed and correlates with a certain polymer concentration in the electrospun solution [55]. PCL and CS are polymers with significantly different chemical properties and finding a common solvent to create a film was an important challenge. Additionally, it was crucial to maintain the optimal viscosity in order to create the double porous membrane structure. By increasing the chitosan ratio, it was possible to electrospin PCL/chitosan blends, and SEM pictures revealed that as the chitosan ratio increased, the fiber diameter and dispersion reduced. According to Roozbahani, Fatemeh et al., PCL-treated chitosan nanofibers with a 70/30 ratio have a smaller average diameter of 205 nm than blended nanofibers made from untreated chitosan, which has a 356 nm diameter [56].

### 3.3. FTIR Analysis

**Figure 3:**
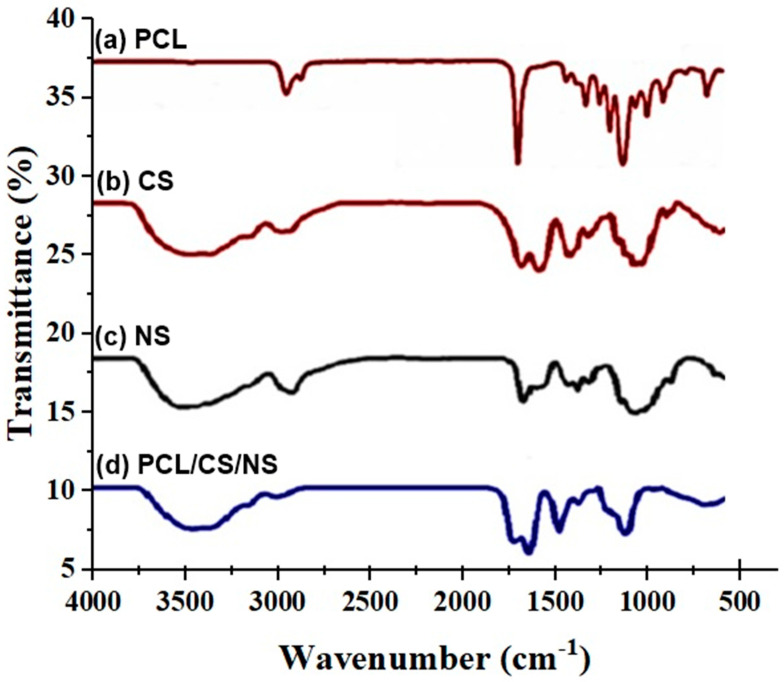
FTIR spectrum of PCL/CS/NS, PCL, CS, and NS extract.

**Figure 4:**
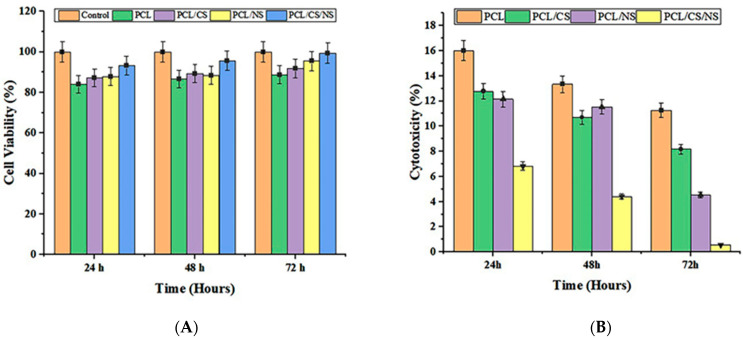
(**A**) L929 cell viability percentages during different periods of time (24, 48, and 72 h). (**B**) L929 cytotoxicity percentages during different periods of time (24, 48, and 72 h).

FTIR analysis was used to determine how electrospinning altered the parts that made up the PCL/CS/NS. The spectra of the PCL/CS/NS are shown in Figure 3. Prior research [57] revealed that the asymmetric and symmetric CH2 stretching peaks of the pure PCL membrane were at 2955 cm^−1^ and 2875 cm^−1^, the CO stretching peak was at 1735 cm^−1^, the asymmetric COC stretching peak was at 1260 cm^−1^, and the symmetric CH2 stretching peak was at 1164 cm^−1^ (CC stretching). The broad peak at 3451 cm^−1^ that distinguished PCL from the PCL/CS/NS spectrum was caused by the stretching vibration of -OH and -NH2 from CS [58]. However, the PCL/CS/NS composite membrane’s peaks were increased to 1500 cm^−1^ as a result of the peaks in the visible region of Figure 4 that belonged to NS [59]. This demonstrated that bioactive materials were electrospun into the PCL/CS/NS composite.

### 3.4. Water Contact Angle and Porosity Results

Wettability is an essential factor to consider when choosing a wound dressing since it influences cell adherence, proliferation, and the ability to absorb exudates. The water contact angle can be used to determine the wettability of a surface. The water contact angle was measured to determine the behavior of the composite PCL/CS/NS mats and to assess the hydrophilicity alterations in the nanocomposite scaffolds. As presented in Table 2, the PCL film exhibited poor hydrophilicity, with an average contact angle of 122.5°, which was in line with the hydrophobic nature of the polymer.

The contact angle value of PCL at 8% decreased to 99.4°, and then to 53.2°, after the addition of CS at 2% and NS at 10%, respectively. Results of the wettability test demonstrated that the incorporation of NS and CS within the PCL matrix may have produced some hydrophilic groups, such as NH and OH, on the surfaces of the nanocomposite membranes. The results of the mechanical properties are displayed in Table 3. The PCL/CS/NS nanofiber mat’s tensile strength was 5.4 ± 0.2 MPa, which was higher than the range of 1.8 ± 0.1 MPa for the PCL nanofibers alone, and was in line with earlier studies [60].

### 3.5. In Vitro Cell Culture Studies

**Figure 5:**
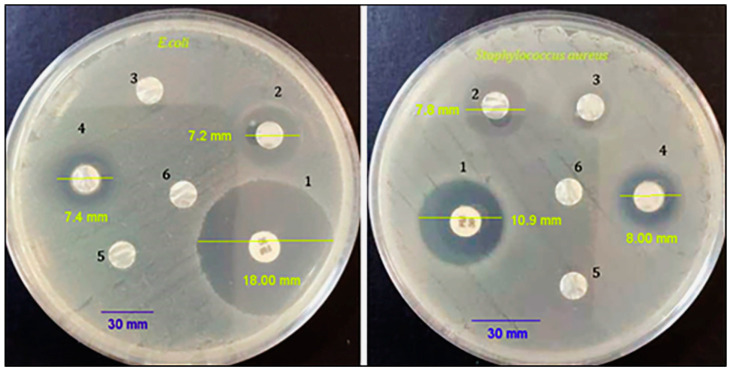
Antimicrobial activity of PCL/CS/NS nanofibers against *S. aureus* and *E. coli*. Inhibition zones (mm): 1—antibiotic; 2—CS/NS; 3—PCL/CS; 4—PCL/CS/NS nanofibers; 5—PCL; 6—negative control (D.W.).

The MTT test was used to determine the impact of PCL/CS/NS nanofiber scaffolds on the viability of L929 cells. The viability of the PCL/CS/NS scaffolds is shown in Figure 5 at 24, 48, and 72 h. As shown in the graph, the growth rate of the PCL/CS/NS nanocomposite scaffold was significantly higher than that of PCL, PCL/CS, and PCL/NS, and it approached that of the control sample by the end of the third day. The PCL/CS/NS scaffold’s fibers had very small diameters compared to pure PCL fibers, providing an appropriate space for cells to be placed. Furthermore, according to the test for determining scaffold hydrophilicity, adding NS to the polymer solution significantly increased the scaffold’s hydrophilicity, resulting in better cell adhesion to the scaffold. In a study conducted by Zagórska-Dziok, Martyna et al., *N. sativa* was found to have no cytotoxic effect on keratinocytes and fibroblasts, at concentrations of 1–1000 μg/mL [61]. Given these findings, it is reasonable to conclude that the PCL/CS/NS scaffold, at the ratio of 3/1/2, is a good option for cell culture because it increased the rate of proliferation of L929 cells over time [62]. This is consistent with the findings of Uddin et al. 2022 [63], who proved that, according to their MTT results, NS-containing composite mats were non-cytotoxic and increased fibroblast migration and proliferation.

### 3.6. Antibacterial Activity

**Figure 6:**
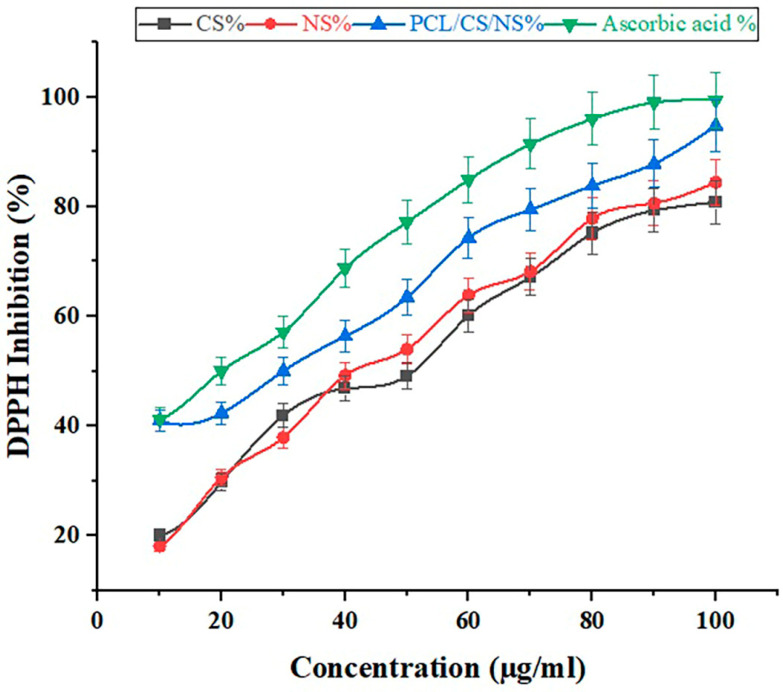
Percentage inhibition of DPPH radical in the presence of different concentrations of CS, NS, PCL/CS/NS nanofiber mats, and ascorbic acid.

An antibacterial evaluation was conducted using a disk diffusion technique for each bacterium. The diameter of the inhibition zone was measured after 24 h of incubation by a caliper. As shown in Figure 6, the prepared PCL/CS nanofiber mats containing NS had better antibacterial properties than PCL and PCL/CS. The results of the current study showed a notable inhibition zone of 8.00 ± 0.22 mm and 7.4 ± 0.16 mm for *S. aureus* and *E. coli,* respectively. The inhibition zone diameter was used as an index of the scaffold’s antibacterial activity in the disk diffusion test; the inhibition zone diameter of the mats containing PCL/CS/NS against *S. aureus* (Gram-positive) was greater than for *E. coli* (Gram-negative bacteria). Ciprofloxacin at 10 µg/mL was used as a positive control. According to the antibacterial activity test results of the present study, the inclusion of NS in the composite scaffold promoted antibacterial activity. Gram-positive bacteria are sensitive to these mats, and these results were in agreement with a result previously reported by Shahverdi et al. 2022 [64]. The antibacterial action of *N. sativa* seed extract may cause bacterial cell membranes to become permeable, resulting in cell destabilization and death [65]. Gram-negative bacteria are more resistant because their cell membranes are double-layered, as opposed to Gram-positive bacteria’s single-layer membranes [66,67].

### 3.7. Antioxidant Activity

Figure 6 depicts the 2,2-diphenyl-1-picrylhydrazyl (DPPH) method used to assess the antioxidant properties of the prepared nanofibrous mats. The free radical scavenging capacities were measured using the DPPH assay. Scavenging is most effective with electron or hydrogen donor scaffolds that quench and stabilize DPPH to DPPH-H. NS-containing scaffolds demonstrated dose-dependent scavenging potency comparable to ascorbic acid (*p* ≤ 0.005) (Table 4). Large amounts of ROS are produced during inflammation, causing biological damage such as lipid, protein, and nucleic acid degradation, and ultimately cell death, which disrupts the recovery process. The use of antioxidants can significantly aid enzymatic repair and metabolism [68]. Many studies have demonstrated that biogenic nanomaterials are a consistent source of antioxidant activity [69,70].

Previous studies have experimented with various extraction methods, including the DPPH scavenging assay, for extracts of *N. sativa* seeds [71]. In addition to beta-sitosterol, the NS seed extract contains significant amounts of other antioxidants, such as various tocopherol and tocotrienol isomers found in the alpha, beta, gamma, and delta forms. Another study found that an ethanolic extract of *N. sativa* seeds inhibited the DPPH scavenging assay by a higher percentage than a methanolic extract, which inhibited the assay by only 3.77% [72].

The current study’s findings are in excellent agreement with those of other investigations. Arif et al. (2021) [73] found that nanosuspensions of *N. sativa* extracts had the highest free radical scavenging activity of up to 55% at doses of 1000 mg/mL, and the lowest activity of up to 28% at 250 mg/mL. This work demonstrated that the DPPH free radical scavenging activity was greatly enhanced by increasing the quantities of the nanosuspensions and *N. sativa* extracts. The maximal amount of free radical scavenging activity for nanosuspensions of *N. sativa* extract was seen at 500 g/mL, according Ali et al. [74]. Thus, the DPPH free radical scavenging activity was dramatically improved by increasing the quantities of the nanofibrous mat.

## 4. Conclusions

According to the research’s findings, Nigella sativa-loaded PCL/CH electrospun nanofibers formed a new nanofibrous scaffold that was discovered to be non-toxic to skin L929 fibroblast cells. The production method resulted in thin fibers with mean diameters as low as 82 nm, high porosity, promising tensile strength, enhanced hydrophilicity, and biocompatibility. The incorporation of N. sativa into the PCL/CH matrix was supported by the findings of the chemical investigation of the nanofibrous composite by FTIR spectroscopy and structural XRD analysis.

The results of the cell viability test, which showed this formulation’s great bio-compatibility, were supported by the MTT assay. The inclusion of Nigella sativa extract also decreases the diameter of nanofibers. It also improves the antioxidant and antibacterial properties.

## Figures and Tables

**Table 1:** Retention time, phytochemical compounds, and peak area % determined by GC–MS analysis of Nigella sativa seed extract.

Peak No.	Ret. Time	Phytochemical Compounds	Molecular Formula	Molecular Weight	Peak Area %
1	5.603	Glycerin	C3H8O3	92	0.47
2	18.622	Phenol, 2,4-bis(1,1-dimethylethyl)-	C14H22O	206	0.18
3	20.18	Phenol, 2,4-bis(1,1-dimethylethyl)-	C14H22O	206	0.82
4	24.244	Phenol, 2,4-bis(1,1-dimethylethyl)-	C14H22O	206	0.93
5	35.471	Hexadecanoic acid, methyl ester	C17H34O2	270	0.73
6	35.555	7,9-Di-tert-butyl-1-oxaspiro(4,5)deca-6,9-diene-2,8-dione	C17H24O3	276	0.15
7	35.807	Hexadecanoic acid, methyl ester	C17H34O2	270	0.52
8	36.575	l-(+)-Ascorbic acid 2,6-dihexadecanoate	C38H68O8	652	4.24
9	36.823	Pentadecanoic acid	C15H30O2	242	3.73
10	37.284	Hexadecanoic acid, ethyl ester	C17H34O2	270	0.82
11	37.511	Hexadecanoic acid, ethyl ester	C17H34O2	270	0.52
12	39.765	9,12-Octadecadienoic acid (Z,Z)-, methyl ester	C19H34O2	294	5.07
13	39.925	10,13-Eicosadienoic acid, methyl ester	C21H38O2	322	6.46
14	40.066	9-Octadecenoic acid (Z)-, methyl ester	C19H36O2	296	1.89
15	40.573	Methyl stearate	C19H38O2	298	0.23
16	41.125	Octadec-9-enoic acid	C18H34O2	282	47.64
17	41.386	9,12-Octadecadienoic acid, ethyl ester	C20H36O2	308	7.71
18	41.485	9,12-Octadecadienoic acid, ethyl ester	C20H36O2	308	16.71
19	42.183	Heptadecanoic acid, 15-methyl-, ethyl ester	C20H40O2	312	0.85
20	42.26	Heptadecanoic acid, 15-methyl-, ethyl ester	C20H40O2	312	0.32

**Table 2:** Hydrophobicity and electrospinning conditions of the electrospun nanofibers.

Sample	Solutions: Ratio	Contact Angle (°) (Hydrophilicity)	FR (mL/h)	TCD (cm)	Voltage (kV)
PCL	-	122.5° ± 2.0	0.5	20	20
PC L/Cs		99.6° ± 4.0	0.5	20	20
PCL/Cs/NS	70:30	53.2 ± 1.0	0.5	20	20

**Table 3:** Physical properties of the PCL/CS/NS nanofibers after cross-linking (*: *p* < 0.05).

Sample	Ultimate Tensile Strength (MPa)	Contact Angle (°) (Hydrophilicity)
PCL	1.8 ± 0.1	117.5 ± 2.0
PCL/Cs/NS	5.4 ± 0.2 *	121.8 ± 2.0 *
PCL/Cs	3.4 ± 0.1	118.2 ± 2.0

**Table 4:** DPPH inhibition (%) of different concentrations of PCL/CS/NS nanofiber mats.

Concentration (µg/mL)	CS %	NS %	NF %	Ascorbic Acid %
10	20.079	18.108	40.986	41.23
20	29.787	30.63	42.342	50.01
30	41.935	37.903	50	57.14
40	46.825	49.206	56.349	68.74
50	49.107	53.968	63.492	77.18
60	60.15	63.888	74.306	84.94
70	67.123	68.211	79.47	91.41
80	75.159	77.844	83.832	96.01
90	79.289	80.662	87.845	99.01
100	80.829	84.455	94.81865	99.44
Mean	55.02 A	56.48 AB	67.34 AB	76.51 B
SD	20.99 *	22.51 *	19.43 *	21.31 *
LSD (*p* < 0.05) =	19.13			

Different letters between any two groups indicate a significant difference at *p* < 0.05.* CS = Chitosan; NS = *Nigella sativa*; NF = PCL/CS/NS nanofibers.

## Data Availability

The datasets used and/or analyzed during the current study are available from the corresponding author on reasonable request.
